# Pulmonary sarcomatoid carcinoma: A rare case report, diagnostic dilemma and review of literature

**DOI:** 10.1097/MD.0000000000038797

**Published:** 2024-07-05

**Authors:** Xilin Liu, Lixin Guo, Xiangfu Ding, Zhichen Kang

**Affiliations:** a Department of Hand and Foot Surgery, China-Japan Union Hospital of Jilin University, Changchun, China; b Department of Rehabilitation, The Second Hospital of Jilin University, Changchun, China; c Department of Thyroid Surgery, The Second Hospital of Jilin University, Changchun, China.

**Keywords:** chemotherapy, diagnosis, disease attributes, lung neoplasms

## Abstract

**Rationale:**

Pulmonary sarcomatoid carcinoma (PSC), a rare tumor, comprises 0.1% to 0.4% of all malignant lung tumors. Given the rarity of PSC, its clinical course, therapeutic guidelines, and patient outcomes remain largely unknown. Therefore, it is imperative to alert clinicians to this extremely rare and instructive early-onset cancer.

**Patient concerns:**

This report describes a 28-year-old woman with PSC, who was initially misdiagnosed with Whipple’s disease. A conclusive diagnosis of PSC was made following careful clinical examination, imaging, and histopathological evaluation of the patient’s biopsy sample. Radiological imaging revealed multiple nodules and mass formations in the left upper lobe of the patient’s lung, with the largest measuring of 5.4 × 3.2 cm.

**Diagnosis:**

Histopathological examination indicated the presence of a malignant neoplasm associated with necrosis suggestive of sarcoma, which was pathologically staged as cT4N1M1.

**Interventions and outcomes:**

A regimen of doxorubicin and ifosfamide was administered therapeutically, resulting in a stable disease state.

**Lessons:**

The rarity and tumor origin challenge the diagnosis, which emphasizes the imperative role of histological examination, immunohistochemistry, and flow cytometry in achieving an accurate diagnosis. This report summarizes the existing publications to provide a comprehensive overview of PSC, including its clinical manifestations, radiographic imaging, pathologic features, diagnostic challenges, treatment strategies, and prognosis, and aims to improve the understanding of PSC.

## 1. Introduction

Pulmonary sarcomatoid carcinoma (PSC) is a rare malignancy that presents a significant clinical challenge owing to its aggressive behavior and poor differentiation.^[1,2]^ The poor prognosis of this disease highlights the urgent need for an enhanced understanding and novel treatment strategies. Ongoing advances in molecular biology techniques have led to the discovery of numerous molecular targets that have, in turn, improved the efficacy of targeted therapies. Expanding our understanding of PSC, particularly its clinical presentation and molecular mechanisms, is crucial for improving clinical outcomes. PSC, a non-small cell lung cancer (NSCLC) characterized by sarcoma-like cells or sarcoma differentiation, accounts for 0.1% to 0.4% of all lung malignancies.^[3,4]^ Notably, PSC is characterized by high malignancy, poor prognosis, and shorter average survival period than other types of NSCLC.^[5]^ Surgical intervention is the treatment of choice for patients who are resistant to chemotherapy and radiotherapy. However, many patients are diagnosed at an advanced stage, rendering them unsuitable for surgery. Moreover, high post-surgical recurrence rates challenge the management of PSC. However, the potential impact of targeted therapies on PSC remains unclear and scarcely reported. Hence, this study aimed to retrospectively investigate the clinical data, pathological characteristics, and molecular and immune phenotypes of a patient diagnosed with PSC and to expand our understanding of this rare disease.

## 2. Case report

**Figure 1.**
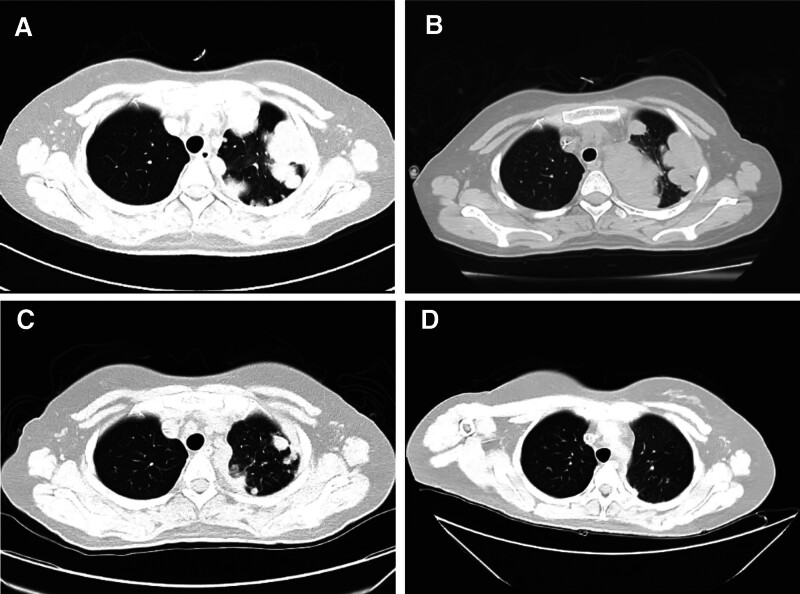
Computerized tomography (CT) scan results. (A) Initial chest CT revealed multiple high-density nodular shadows within the left lung and pleura. (B) A follow-up examination after 7 days demonstrated an increased occupation within the left lung, with the largest mass measuring of 5.2 × 3.6 cm.(C) A subsequent chest CT demonstrated a significant reduction in the high-density shadow range within the left upper lobe compared to the initial examination. (D) A follow-up chest CT revealed multiple varied-sized nodular with high-density shadows in the left upper lobe, some of which exhibited undefined boundaries with the pleura. The size and number of nodules significantly decreased and some of them exhibited cavitations.

**Figure 2.**
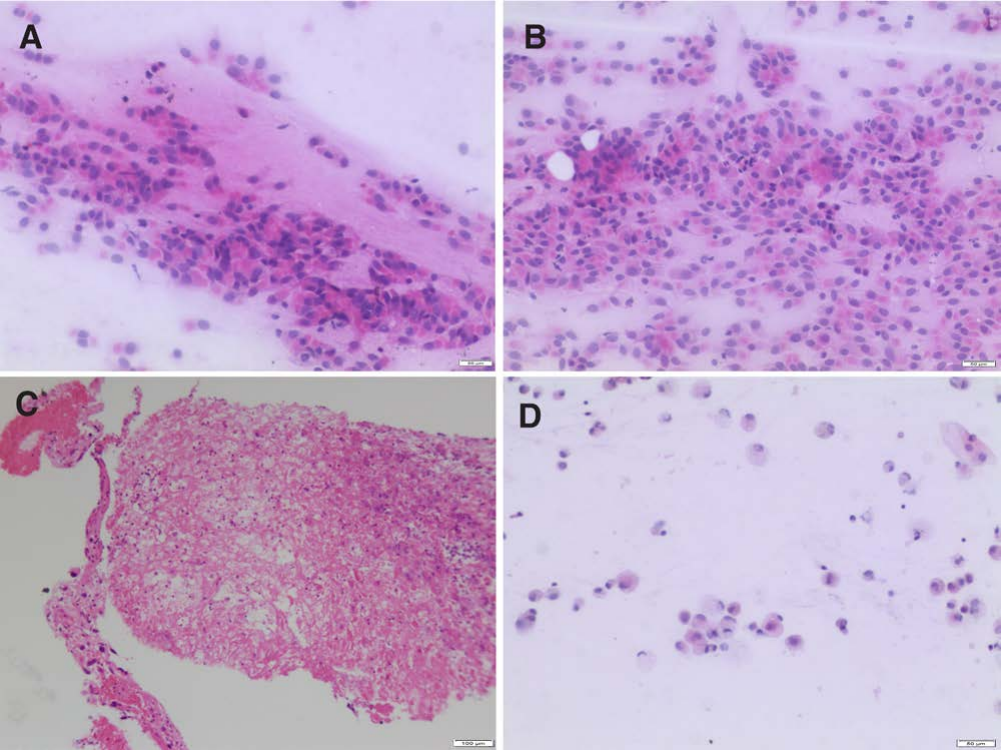
Cytological and histological examination results. (A, B) Cytological examination of bronchial brushing demonstrated that there were lots of epithelial cells and only a few lymphocytes (magnification: ×400). (C) The results of the needle biopsy specimens (H&E, magnification: ×100) revealed fibrinoid necrosis along with minor amounts of neutrophils, lymphocytes, and nuclear heterogeneous cells. (D) Bronchial brushing showed a few of squamous epithelial cells, tissue cells, and epithelial cells (magnification: ×400).

The patient, a 28-year-old female, began to experience dry cough, intermittent left back and rib area pain, and fever peaking at 39.1°C on October 1, 2022. Notably, the patient did not exhibit symptoms such as nausea, vomiting, abdominal pain, bloating, or diarrhea. Initial chest computed tomography (CT) revealed multiple high-density nodular shadows within the left lung and pleura (Fig. 1A). Two bronchoscopies were performed, and the results suggested Whipple’s disease (Fig. 2). Subsequently, a CT-guided lung puncture was performed to obtain a biopsy sample; however, the results were inconclusive. A PET-CT examination subsequently identified multiple nodules and masses in the left lung and pleura, with the largest measuring of 5.4 × 3.2 cm and SUVmax of 5.4. Additionally, noticeable enlargement and increased metabolism of the lymph nodes in the interlobar area of the mediastinum, left pulmonary hilum, and left superior lobe were detected. The patient received anti-infection treatment for 7 days, and a follow-up CT examination demonstrated an enlarged nodule within the left lung, with the largest measuring 5.2 × 3.6 cm (Fig. 1B). Moreover, new subpleural nodules were observed in the outer basal segment of the left lung lower lobe. Despite the anti-infection treatment, the patient’s symptoms persisted, and enhanced CT indicated the largest nodule, measuring 5.0 × 3.8 cm.

**Figure 3.**
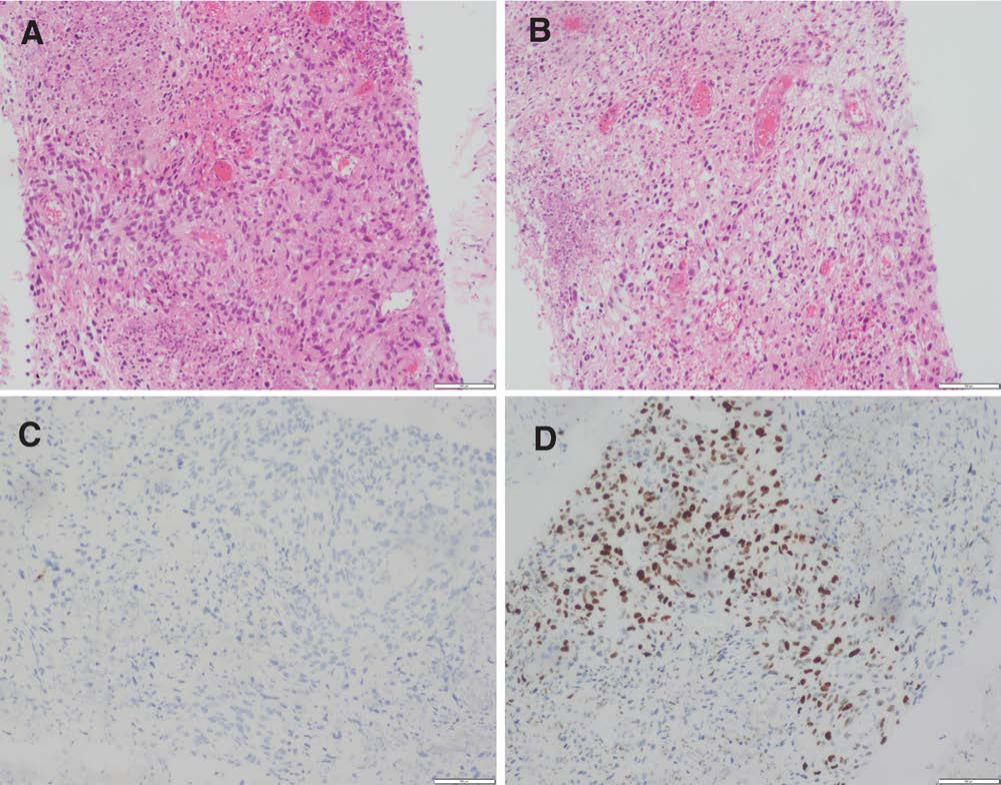
Histopathological and Immunohistochemical findings (magnification: ×100). (A, B) Histopathological examination indicated malignant tumors accompanied by necrosis, leading to a consideration of sarcoma. (C) Immunohistochemical staining for CK. (D) Immunohistochemical staining for Ki-67. CK = cytokeratins.

Histopathological examination suggested malignant tumors accompanied by necrosis, leading to a diagnosis of sarcoma. The immunohistochemical findings were: PD-L1 (TPS < 1%), CK7 (-), P40 (-), HMB45 (-), S-100 (-), CD34 (-), CD138 (-), KI-67 (+, 4%–6%) (Fig. 3). Based on these findings, the patient was pathologically diagnosed with cT4N1M1. No abnormal lymphocyte phenotype was detected by flow cytometry, and the proportion of lymphocytes was relatively reduced, which consisted of 55.93% T cells (CD4: CD8 = 1.46), 24.68% NK cells, and 13.12% mature B cells (which were polyclonal). No significant abnormalities were detected in any of these results. The patient was then treated with a combination of doxorubicin (DOX) and ifosfamide (IFO) (AI regimen), a standard first-line treatment for metastatic soft tissue sarcoma. A subsequent chest CT examination on February 1, 2023, demonstrated a significant reduction in the high-density shadow within the left upper lung lobe compared with the initial examination (Fig. 1C). Follow-up chest CT after 3 months revealed multiple varied-sized nodular high-density shadows in the left upper lung lobe, some of which exhibited undefined boundaries with the pleura (Fig. 1D). The size and number of nodules significantly decreased with cavitation in some cases. After completion of 5 cycles of chemotherapy without any associated adverse reactions, the patient’s condition stabilized. This study was approved by the Ethics Committee of the Second Hospital of Jilin University. Written informed consent was obtained from all participants prior to the study.

## 3. Discussion

PSC is a rare type of lung cancer, with a significantly lower incidence and poorer prognosis than other NSCLCs.^[6]^ The concept of sarcomatoid differentiation was first proposed by Kontic et al in 1865, suggesting heterogeneous differentiation of a single primitive cell through both epithelial and mesenchymal stages during the early phase of tumor transformation.^[7]^ The 2004 WHO classification of lung cancer defines PSC as a class of poorly differentiated NSCLC exhibiting sarcoma or sarcoma-like differentiation (striate and/or giant cells), which includes 5 subtypes (pleomorphic carcinoma, spindle cell carcinoma, giant cell carcinoma, carcinosarcoma, and pulmonary blastoma).^[8]^ Retrospective case analyses show that PSC has a rapid clinical course, is highly invasive, resistant to chemotherapy drugs, and is associated with poor prognosis.^[9,10]^ Recently, with the development of new technologies, such as immunohistochemistry and electron microscopy, it has been confirmed that sarcoma-like cancer is a malignant tumor with a carcinoma-like morphology, and the sarcoma-like components are derived from cancer.^[11]^ The histological heterogeneity of PSC determines the complexity and difficulty of its diagnosis from subtypes, other cancers, or sarcomas.^[12,13]^

PSC has a low incidence rate and there are few reports in the literature. Its tissue is mixed with sarcoma components with poor differentiation, and its clinical manifestations are similar to those of other types of lung cancer. It is difficult to diagnose early, and PSC can easily metastasize. Currently, the efficacy of adjuvant radiotherapy and chemotherapy for patients with metastasis is unclear. Most studies suggest a low sensitivity of PSC to radiotherapy and chemotherapy and a poor prognosis. Rossi et al^[14]^ suggested that pathological staging of PSC is correlated with prognosis. The clinical and pathological characteristics of PSC are obvious, and postoperative pathological examination and immunohistochemical staining are helpful for the diagnosis.

PSC is prone to misdiagnosis as inflammatory pseudotumors, synovial sarcoma, and connective tissue proliferative carcinoma. For inflammatory pseudotumors, spindle cells, short spindle cells, and a large number of inflammatory cells can be found in the tissue, and immunohistochemical staining shows CD68- and SMA-positive cells. Synovial sarcoma can occur in any tissue or organ, and is a malignant tumor with bidirectional differentiation characteristics. Immunohistochemical staining revealed cytokeratins (CK), epithelial membrane antigen (EMA), and CD99 positivity. For connective tissue proliferative carcinoma, fibrous tissue proliferation can be found around the cancer nest or accompanied by partial glassy degeneration, negative epithelial markers, and stromal fibroblasts without malignant cell characteristics. Sarcoma-like components in PSC express epithelial markers such as CK and EMA, while cancer cells express mesenchymal markers such as vimentin.

Primary Pulmonary Sarcoma (PPS) is a rare type of non-epithelial malignant tumor of the lung, accounts for 0.013% to 1.1% of malignant tumors.^[15,16]^ The incidence rate of PPS is rare in primary lung malignancies.^[17,18]^ The clinical manifestations of PPS are similar to those of other lung malignancies, and are mainly characterized by cough, sputum production, chest pain, and chest tightness, making it easy to misdiagnose other lung or mediastinal tumor diseases. Once the tumor has a large volume and invades externally, symptoms such as hemoptysis and difficulty breathing may appear. PPS is difficult to diagnose and presents as a smooth circular or quasi-circular lobulated mass on imaging. The mass usually shows expansive growth with uniform density, and a few lesions are accompanied by signs of spiculation. PPS rarely invades the lymph nodes, but pleura, once the tumor approaches it, causes pleural effusion. The precise diagnosis of PPS depends on the pathology, cell morphology, and immunohistochemistry. PPS rarely invades the tracheal epithelium, and the rate of shed cells is low. Both bronchoscopy and percutaneous lung biopsy have a high misdiagnosis rate owing to the small amount of tissue obtained. PPS can be easily misdiagnosed as PSC under light microscopy, enhancing the neccessarity to distinguish between epithelial origin and mesenchymal components based on immunohistochemistry,^[8,19]^ including Vimentin, Desmin, CK, EMA, S-100 proteins. Vimentin is a mesenchymal tumor marker with high sensitivity and specificity and can be used in conjunction with CK to distinguish between most epithelial and mesenchymal tumors.^[20]^

Previous studies have shown that PSC are prone to invade the pleura,^[13,21]^ which was also observed in this case. The presence or absence of parietal pleural involvement is an independent prognostic factor that indicates the survival rate of patients with PSC.^[13,21]^ Due to the high invasiveness of PSC, early occurrence of intra- and extrapulmonary metastasis occurs, leading to the loss of the opportunity for surgery at the time of diagnosis, resulting in a lower overall prognosis of PSC than that of other NSCLC.^[22]^

The appearance of PSC on computed tomography (CT) has certain characteristics: mostly a single tumor, central or peripheral, circular, with uniform density and smooth edges, burrs or lobulation, and rare calcification. Enhanced CT shows circular or patchy enhancement^[23,24]^ with signs of chest wall invasion and/or pleural effusion.^[25]^

Previous studies found that PSC has a higher average SUVmax value than ordinary NSCLC (PSC group vs ordinary NSCLC group, 15.11 vs 7.66) in PET-CT.^[26]^ Clinically, most patients with PSC are diagnosed at an advanced stage and it is difficult to obtain sufficient surgical specimens. Therefore, there is an urgent need to determine the diagnosis based on a small number of specimens. Pelosi et al^[27]^proposed a modified vimentin histological score (M-VHS), which was roughly equivalent between biopsy specimens and surgical specimens. Therefore, M-VHS has value for diagnosis using small biopsy specimens.

The imaging manifestations of primary pulmonary sarcomatoid carcinoma depend on the expression and ratio of the sarcoma component to the cancer component, which includes the characteristics of both sarcoma and cancer. In this case, based on the data from plain CT and PET-CT, the imaging features are summarized as follows: multiple enlarged lymph nodes with increased glucose metabolism were found in the left hilum and upper lobe of the lung, with the shortest diameter of 9.0 mm. Multiple masses and nodules with increased glucose metabolism were found in the left upper lobe of the lung and left pleura, with the largest measuring of 5.4 × 3.2 cm and SUVmax 5.4. Higher glucose metabolism activity was found in the left lung apex, with a size of 5.4 × 3.2 cm and SUVmax of 9.7.

The malignancy and nonspecific clinical manifestations of PSC are related to the location and affected structure of the lungs. For example, when tumors affect the main bronchus, mediastinum, and chest wall, clinical manifestations such as difficulty in breathing and pleural effusion may occur.^[28]^ A precise diagnosis must be made based on clinical features, immunohistochemistry, and molecular biology findings. Stage is determined by tumor size, lymph node status, and the presence of metastasis (TNM). It is easy to misdiagnose based on small biopsy or cytological pathological examination, and the WHO recommends obtaining sufficiently large surgical specimens combined with immunohistochemistry and microscopy to assist in diagnosis.^[28]^

### 3.1. Clinical characteristics of PSC

PSC tends to be more common in elderly men, especially those who are smokers.^[9,14,28–30]^ PSC is highly invasive and can metastasize to various tissues through lymphatic and hematogenous spread. Its metastatic patterns are similar to those of traditional NSCLC, with a predisposition towards the brain, bone, adrenal, and liver,^[31–33]^ and a typical peritoneal metastasis.^[34]^ The radiological characteristics of PSC on chest X-rays are nonspecific and resemble those of other types of lung cancer.^[35]^ However, an enhanced CT scan may reveal certain differences such as irregular or annular enhancement around the mass and relatively non-enhancing central areas, indicating necrosis due to hemorrhage. PSC can be easily confused with benign lung diseases such as tuberculosis and pneumonia in the early stages. Peripheral PSC typically manifest as large masses that grow relatively quickly and easily invade the mediastinal diaphragm, adjacent pleura, chest wall, intrapulmonary blood vessels, and other structures. Liquefied necrosis and cancerous cavities can be found in the central center of the tumor owing to insufficient blood supply.^[35]^ The prognosis of patients with PSC is worse than that of patients with traditional NSCLC, largely due to resistance to conventional treatments and rapid recurrence after surgical resection.^[10,25,34,36]^ However, some studies also suggested that the prognosis of patients with PSC is not significantly different from that of other patients with NSCLC.^[37,38]^ Owing to its low incidence, there is no standardized treatment for PSC. Previous studies indicate that the overall survival rate (OS) of PSC patients is not significantly associated with factors such as race, age, sex, smoking history, tumor size, pleural invasion, or lymph node metastasis,^[29,39,40]^ but is dependent on histological subtype, tumor stage, distant metastases, pathological stage, vascular invasion, and mutations in genes such as programmed cell death-ligand 1(*PD-L1), TLG-P*, and *KRAS*.^[1,22,39–43]^

### 3.2. Diagnosis of PSC

PSC often manifests as either central or peripheral lung lesions, with a pre-direction to the upper lobes of the lungs.^[32,44,45]^ Pleomorphic carcinoma and pulmonary blastoma are mostly located in the peripheral area of the lungs, with chest wall involvement in pleomorphic carcinoma.^[28,46]^ Intrabronchial invasion can be found in PSC, which is most common in carcinosarcomas and rare in pleomorphic carcinoma.^[28,44,47]^ PSC grow and invade bronchial trees, the lung parenchyma, and adjacent anatomical structures. Tumors may have distinct or indistinct borders, with extensive necrosis and bleeding. In addition, they may appear as round or large masses with a soft texture or varying degrees of firmness.^[32]^

PSC needs to be distinguished from other types of NSCLC, carcinosarcomas, and sarcomas^[48]^ with the assistance of immunohistochemistry.^[14,37,46,49–51]^ Immunohistochemical staining of PSC often shows coexisting epithelial and interlobular markers, which result from epithelial-mesenchymal transition (EMT). Currently, the widely used immunohistochemical indicators include 2 categories: stromal cell biological markers, including desmin, vimentin, and CD68, and epithelial biological markers, including CK, anti-pan-cytokeratin antibody (AE1/AE3), EMA, and carcinoembryonic antigen.^[41,52]^

Due to its low incidence, the awareness of PSC is insufficient and it is easy to misdiagnose. Furthermore, preoperative pathological diagnoses, such as sputum cytology, bronchoscopic biopsy, and CT-guided percutaneous needle lung biopsy, are not definitive because of tissue insufficiency.^[53]^ Sun et al^[53]^ analyzed the characteristics of 55 patients with PSC and found that 8 cases were diagnosed through fiberoptic bronchoscopy, 26 cases were diagnosed through CT-guided lung biopsy, and 21 cases were diagnosed through pathological examination after surgical resection of the lesion, which indicates that it is difficult to diagnose PSC through puncture biopsy. In this case, the patient experienced multiple metastases, which made it less likely to choose surgery and it was difficult to obtain sufficient specimens. PSC needs to be distinguished from the following diseases^[28,54]^: primary or metastatic monophasic and biphasic (composed of epithelioid cells and spindle cells) sarcomas (i.e. synovial sarcoma, malignant solitary fibroma, inflammatory myofibroblastoma), sarcomatoid mesothelioma, spindle cell melanoma, sarcomatoid thymic carcinoma, and choriocarcinoma, etc.

Whipple’s disease is also a rare disease, with an incidence of approximately 1/10,000,000. The average age at diagnosis is approximately 55 years, of which 85% are male.^[55]^ The most common symptoms of Whipple’s disease are joint pain, diarrhea, fatty diarrhea, weight loss, lymph node enlargement, abdominal pain, hypoalbuminemia, and anemia.^[56]^ As the disease progresses, patients may experience symptoms of the central nervous and cardiovascular systems. Approximately 10% to 25% of patients have no digestive symptoms, known as “dry Whipple’s disease.”^[57]^ A few patients with Whipple’s disease have typical clinical manifestations,^[55]^ and the respiratory system can manifest with dry cough, low fever, fatigue, chest pain, and difficulty breathing.

Whipple’s disease affects multiple tissues and organs, leading to a high susceptibility to misdiagnosis and mistreatment, resulting in serious adverse consequences. Patients with Whipple’s disease generally show nonspecific laboratory test results. In most cases, routine blood tests showed increased white blood cell count, mainly neutrophils, and increased inflammatory indicators, such as increased erythrocyte sedimentation rate, elevated C-reactive protein, and decreased hemoglobin content. Blood biochemical examinations showed decreased serum albumin, cholesterol, carotene, and iron levels. Fecal examinations revealed positive occult blood test results and the presence of fat in the stool. A mild increase in the white blood cell count and protein content was found in the cerebrospinal fluid. Rheumatoid factor and antinuclear antibody test results were negative. Immunohistochemical techniques can detect the presence of Tropheryma whipplei (TW) in macrophages in various tissues by using a specific anti-TW antibody under an optical microscope.^[56,58]^ Currently, using PCR to detect the amplification of the 16S rRNA gene is the preferred molecular biology method for diagnosing Whipple’s disease, with higher sensitivity and specificity than other methods.^[58]^ In this case, after evaluating the results from histopathological and clinical examinations and imaging techniques, a diagnosis of PSC was made.

Flow cytometry is widely used for exploratory immune profiling and biomarker discovery in cancers and other diseases. The chromosomal content of solid tumors, as reflected by the deoxyribonucleic acid content, can now be rapidly and reliably measured by flow cytometry. Flow cytometric detection of cytokeratin-positive cells within lymph nodes has better sensitivity than conventional histological staining and approximately equal sensitivity to that of immunohistochemical staining.^[59]^ Flow cytometry has emerged as a key tool for profiling multiple parameters of the immune system, including vital functional and exhausting markers associated with immune response quality.^[60]^ However, flow cytometry is limited by the number of parameters that can be analyzed simultaneously, which severely restricts its utility. Flow cytometry is relatively insensitive in measuring deoxyribonucleic acid abnormalities but is useful in detecting relatively large and clonal populations.^[61]^ Flow cytometry requires large sample sizes to cover diverse immune subsets, which is particularly detrimental to tumor biopsies with limited sample sizes.

### 3.3. Treatment of PSC

Surgical resection is the primary treatment strategy for patients with early stage NSCLC. However, retrospective studies have shown that patients with PSC treated with surgical resection have poorer overall clinical outcomes than patients with other types of NSCLC.^[6,10,33,36,62]^ A low incidence of PSC has led to a lack of controlled randomized clinical trials to develop suitable chemotherapy strategies. Bae et al reported that although patients received first-line, cisplatin-based, dual-drug chemotherapy, 85% of these patients (11 out of 13) still experienced disease progression. The median overall survival rate from the initiation of first-line palliative chemotherapy was only 5 months (range, 2–12 months), with a median follow-up of 16 months.^[63]^

Soft tissue sarcoma is a term used to describe a heterogeneous group of rare tumors. Since the initial description of the activity of doxorubicin, several other agents have been involved in the treatment of these diseases. Despite being 2 recently approved drugs, doxorubicin and ifosfamide remain the most effective chemotherapy drugs for the majority of these tumors. The prognosis for PSC is poor, with some studies reporting a 5-year survival of 24.5%.^[10]^ There is no standardized therapy for PSC with strategies aimed at managing soft tissue sarcomas or other NSCLC. Very little benefit has been found with chemotherapy alone, but some success has been achieved with adjuvant therapy in surgical management.^[64,65]^ The use of doxorubicin plus ifosfamide and pemetrexed has been established for the management of soft tissue carcinoma and nonsquamous NSCLC, respectively.^[66,67]^ Paredes Mogica et al^[68]^ reported the case of a patient diagnosed with PSC managed with a combination of doxorubicin, ifosfamide, and pemetrexed.

The number of studies on palliative chemotherapy for advanced diseases is limited, and the results show that PSC respond poorly to systemic chemotherapy, with limited therapeutic effects.^[63,69]^ PSC, a unique subset of NSCLC, have distinct molecular characteristics. Previous studies have shown that EMT plays a crucial role in initiating and developing malignant tumors,^[70,71]^ and negative regulation of EMT is a therapeutic strategy for sarcoma-like cancer. In addition, many studies have shown that EMT plays an important role in tumor infiltration and metastasis.^[72,73]^ EMT in epithelial tissue may induce the development of PSC. The transformation into an EMT phenotype can loosen the density of intercellular connections, enabling tumor cells to break free from the constraints of intercellular connections, which, in turn, increases the invasive capability of tumor cells. This process is of great significance in the infiltration and metastasis of PSC. The use of EGFR inhibitors has changed the treatment strategies for NSCLC. For patients with advanced NSCLC mutations, epidermal growth factor receptor-tyrosine kinase inhibitors have become an efficient and cutting-edge treatment.^[74]^ Although few patients with PSC receive EGFR inhibitors, it is worth noting that patients with PSC carrying *EGFR* gene mutations lack significant and lasting clinical responses to EGFR inhibitors.^[75,76]^ There is controversy about the probability of EGFR mutations in PSC, which may be caused by the frequency of EGFR mutations in PSC patients of different races, genders, and tumor subtypes. KRAS is one of the most common mutations in non-scale NSCLC and has a high rate in PSC.^[77–80]^ Results from preclinical research on KRAS inhibitors suggest KRAS mutations as a therapeutic target for PSC.^[43,74]^

Recently, immunotherapy has increased rapidly and has broad prospects for development.^[81,82]^ Programmed cell death protein 1 (PD-1), cytotoxic T lymphocyte-associated antigen-4, PD-L1, and PD-L2 are associated with anti-tumor immune responses. The interaction between PD-1 and related ligands inhibits T-cell function and thus causes immune escape.^[83,84]^ Immune checkpoint blockades block this process and play an antitumor role. Studies have shown that PD-L1 expression is elevated in PSC.^[85–88]^ However, the effect of immune checkpoint blockades on PSC has rarely been reported. Currently, most studies on immunotherapy for patients with pulmonary sarcoma-like cancer have been reported on a case-by-case basis. Cimpeanu et al^[89]^ reported one case of a patient with stage III PSC whose PD-L1 expression rate was > 50%. After 5 cycles of pembrolizumab treatment, the tumor shrank by more than 80% compared with the initial tumor. In addition, 14-month progression-free survival period was achieved. Kotlowska et al^[90]^ reported a PSC patient with liver and small intestine metastases at the time of diagnosis. The disease progressed after radiotherapy and chemotherapy. However, after immunotherapy, the abdominal lesion was completely relieved, the primary lung lesion was stable, and the total survival period of the patient was 5 years. The reason for the high PD-L1 expression in PSC remains unclear. Some researchers believe that EMT may play a role,^[85,91]^ while others connect it to KRAS mutation.^[86]^ Given the high expression of PD-L1 in PSC, PD-L1 testing is recommended in patients with PSC to guide treatment and evaluate prognosis.^[42]^ The immunohistochemical results in this case showed a high PD-L1 expression (TPS < 1%).

## 4. Conclusions

PSC is a rare and highly malignant NSCLC subtype, with certain difficulties in preoperative diagnosis and poor prognosis. It is unresponsive to traditional chemotherapy or radiotherapy. Pathological diagnosis and treatment of PSC remains a great challenge. In the present case, the disease was pathologically staged as cT4N1M1. Therapeutically, a regimen of DOX and IFO was administered, and the size and number of nodules significantly decreased according to the follow-up chest CT, which suggested the efficacy of using DOX and IFO in PSC patients. However, more extensive clinical trials must be conducted to determine whether the DOX and IFO combination can be used to treat advanced PSC. With the development of medical science and molecular biology, in-depth study of molecular characteristics and the application of new therapeutic targets and targeted drugs may improve the therapeutic outcomes of PSC. Further research is needed to confirm the benefits of a strategy that targets multiple targets or incorporates immunotherapies. Development of novel and effective treatment strategies is urgently needed for patients with PSC, which could ultimately improve the survival rate and quality of life of patients with this rare and challenging subtype of lung cancer.

## Acknowledgments

We would like to thank the patient and his family.

## Author contributions

**Conceptualization:** Xilin Liu, Lixin Guo, Xiangfu Ding, Zhichen Kang.

**Data curation:** Xilin Liu, Lixin Guo.

**Funding acquisition:** Xilin Liu, Xiangfu Ding.

**Investigation:** Xilin Liu, Lixin Guo.

**Supervision:** Xilin Liu, Xiangfu Ding.

**Writing – original draft:** Xilin Liu.

**Writing – review & editing:** Xilin Liu, Lixin Guo, Zhichen Kang.

## References

[1] Shum E Stuart M Borczuk A Wang F Cheng H Halmos B. Recent advances in the management of pulmonary sarcomatoid carcinoma. Expert Rev Respir Med. 2016;10:407–16. 26962707 10.1586/17476348.2016.1157475

[2] Yvorel V Patoir A Casteillo F. PD-L1 expression in pleomorphic, spindle cell and giant cell carcinoma of the lung is related to TTF-1, p40 expression and might indicate a worse prognosis. PLoS One. 2017;12:e0180346. 28671973 10.1371/journal.pone.0180346 PMC5495439

[3] Travis WD. Pathology of lung cancer. Clin Chest Med. 2002;23:65–81, viii. 11901921 10.1016/s0272-5231(03)00061-3

[4] Brambilla E Travis WD Colby TV Corrin B Shimosato Y. The new World Health Organization classification of lung tumours. Eur Respir J. 2001;18:1059–68. 11829087 10.1183/09031936.01.00275301

[5] Huang S-Y Shen S-J Li X-Y. Pulmonary sarcomatoid carcinoma: a clinicopathologic study and prognostic analysis of 51 cases. World J Surg Oncol. 2013;11:252. 24088577 10.1186/1477-7819-11-252 PMC3850921

[6] Park JS Lee Y Han J. Clinicopathologic outcomes of curative resection for sarcomatoid carcinoma of the lung. Oncology (Huntingt). 2011;81:206–13. 10.1159/000333095 22076573

[7] Kontic M Stojsic J Stevic R Bunjevacki V Jekić B Dobricic V. Could spindle cell lung carcinoma be considered and treated as sarcoma, according to its clinical course, morphology, immunophenotype and genetic finding? Pathol Oncol Res. 2013;19:129–33. 22923000 10.1007/s12253-012-9562-4

[8] Beasley MB Brambilla E Travis WD. The 2004 World Health Organization classification of lung tumors. Semin Roentgenol. 2005;40:90–7. 15898407 10.1053/j.ro.2005.01.001

[9] Travis WD Brambilla E Nicholson AG.; WHO Panel. The 2015 World Health Organization classification of lung tumors: impact of genetic, clinical and radiologic advances since the 2004 classification. J Thorac Oncol. 2015;10:1243–60. 26291008 10.1097/JTO.0000000000000630

[10] Martin LW Correa AM Ordonez NG. Sarcomatoid carcinoma of the lung: a predictor of poor prognosis. Ann Thorac Surg. 2007;84:973–80. 17720411 10.1016/j.athoracsur.2007.03.099

[11] Thomas VT Hinson S Konduri K. Epithelial–mesenchymal transition in pulmonary carcinosarcoma: case report and literature review. Ther Adv Med Oncol. 2012;4:31–7. 22229046 10.1177/1758834011421949 PMC3244200

[12] Terra SBSP Aubry MC Yi ES Boland JM. Immunohistochemical study of 36 cases of pulmonary sarcomatoid carcinoma – sensitivity of TTF-1 is superior to napsin. Hum Pathol. 2014;45:294–302. 24331839 10.1016/j.humpath.2013.09.005

[13] Vieira T Antoine M Ruppert A-M. Blood vessel invasion is a major feature and a factor of poor prognosis in sarcomatoid carcinoma of the lung. Lung Cancer. 2014;85:276–81. 24997135 10.1016/j.lungcan.2014.06.004

[14] Rossi G Cavazza A Sturm N. Pulmonary carcinomas with pleomorphic, sarcomatoid, or sarcomatous elements: a clinicopathologic and immunohistochemical study of 75 cases. Am J Surg Pathol. 2003;27:311–24. 12604887 10.1097/00000478-200303000-00004

[15] Nascimento AG Unni KK Bernatz PE. Sarcomas of the lung. Mayo Clin Proc. 1982;57:355–9. 6952059

[16] Gołota J Osowiecka K Orłowski T. Primary pulmonary sarcoma – treatment outcomes depending on the different types of radical operation. Kardiochir Torakochirurgia Pol. 2019;16:1–6. 31043968 10.5114/kitp.2019.83938 PMC6491372

[17] Sobande F Samayoa LM Moore AR Adams K Reynolds C. Primary pulmonary synovial sarcoma presenting as a breast mass. Breast J. 2011;17:418–9. 21645168 10.1111/j.1524-4741.2011.01094.x

[18] Janssen JP Mulder JJ Wagenaar SS Elbers HR van den Bosch JM. Primary sarcoma of the lung: a clinical study with long-term follow-up. Ann Thorac Surg. 1994;58:1151–5. 7944769 10.1016/0003-4975(94)90476-6

[19] Hartel PH Fanburg-Smith JC Frazier AA. Primary pulmonary and mediastinal synovial sarcoma: a clinicopathologic study of 60 cases and comparison with five prior series. Mod Pathol. 2007;20:760–9. 17464314 10.1038/modpathol.3800795

[20] Liu K Li W. Analysis of 19 cases of primary pulmonary sarcoma. Zhongguo Fei Ai Za Zhi Chin J Lung Cancer. 2012;15:375–80. 10.3779/j.issn.1009-3419.2012.06.09 PMC6000307 22681925

[21] Gu L Xu Y Chen Z Pan Y Lu S. Clinical analysis of 95 cases of pulmonary sarcomatoid carcinoma. Biomed Pharmacother. 2015;76:134–40. 26653560 10.1016/j.biopha.2015.10.009

[22] Maneenil K Xue Z Liu M. Sarcomatoid carcinoma of the lung: the Mayo clinic experience in 127 patients. Clin Lung Cancer. 2018;19:e323–33. 29454534 10.1016/j.cllc.2017.12.008

[23] Xu X Song W Sui X. Computed tomographic and pathological features of primary pulmonary sarcomatoid carcinoma. Zhongguo Yi Xue Ke Xue Yuan Xue Bao. 2016;38:93–8. 26956864 10.3881/j.issn.1000-503X.2016.01.017

[24] Ouziane I Boutayeb S Mrabti H Lalya I Rimani M Errihani H. Sarcomatoid carcinoma of the lung: a model of resistance of chemotherapy. N Am J Med Sci. 2014;6:342–5. 25077084 10.4103/1947-2714.136920 PMC4114013

[25] Ung M Rouquette I Filleron T. Characteristics and clinical outcomes of sarcomatoid carcinoma of the lung. Clin Lung Cancer. 2016;17:391–7. 27105684 10.1016/j.cllc.2016.03.001

[26] Rapicetta C Lococo F Stefani A. Primary sarcomatoid carcinoma of the lung: radiometabolic (18F-FDG PET/CT) findings and correlation with clinico-pathological and survival results. Lung. 2016;194:653–7. 27300448 10.1007/s00408-016-9904-1

[27] Pelosi G Melotti F Cavazza A. A modified vimentin histological score helps recognize pulmonary sarcomatoid carcinoma in small biopsy samples. Anticancer Res. 2012;32:1463–73. 22493387

[28] Pelosi G Sonzogni A De Pas T. Review article: pulmonary sarcomatoid carcinomas: a practical overview. Int J Surg Pathol. 2010;18:103–20. 19124452 10.1177/1066896908330049

[29] Rahouma M Kamel M Narula N. Pulmonary sarcomatoid carcinoma: an analysis of a rare cancer from the Surveillance, epidemiology, and end results database. Eur J Cardiothorac Surg. 2018;53:828–34. 29240878 10.1093/ejcts/ezx417

[30] Steuer CE Behera M Liu Y. Pulmonary sarcomatoid carcinoma: an analysis of the National cancer data base. Clin Lung Cancer. 2017;18:286–92. 28043773 10.1016/j.cllc.2016.11.016

[31] Mochizuki T Ishii G Nagai K. Pleomorphic carcinoma of the lung: clinicopathologic characteristics of 70 cases. Am J Surg Pathol. 2008;32:1727–35. 18769330 10.1097/PAS.0b013e3181804302

[32] Berho M Moran CA Suster S. Malignant mixed epithelial/mesenchymal neoplasms of the lung. Semin Diagn Pathol. 1995;12:123–39. 7638446

[33] Davis MP Eagan RT Weiland LH Pairolero PC. Carcinosarcoma of the lung: mayo clinic experience and response to chemotherapy. Mayo Clin Proc. 1984;59:598–603. 6381913 10.1016/s0025-6196(12)62410-0

[34] Vieira T Girard N Ung M. Efficacy of first-line chemotherapy in patients with advanced lung sarcomatoid carcinoma. J Thorac Oncol. 2013;8:1574–7. 24389441 10.1097/01.JTO.0000437008.00554.90

[35] Kim TH Kim SJ Ryu YH. Pleomorphic carcinoma of lung: comparison of CT features and pathologic findings. Radiology. 2004;232:554–9. 15215543 10.1148/radiol.2322031201

[36] Yuki T Sakuma T Ohbayashi C. Pleomorphic carcinoma of the lung: a surgical outcome. J Thorac Cardiovasc Surg. 2007;134:399–404. 17662779 10.1016/j.jtcvs.2007.04.018

[37] Nakajima M Kasai T Hashimoto H Iwata Y Manabe H. Sarcomatoid carcinoma of the lung: a clinicopathologic study of 37 cases. Cancer. 1999;86:608–16. 10440688

[38] Pelosi G Fraggetta F Nappi O. Pleomorphic carcinomas of the lung show a selective distribution of gene products involved in cell differentiation, cell cycle control, tumor growth, and tumor cell motility: a clinicopathologic and immunohistochemical study of 31 cases. Am J Surg Pathol. 2003;27:1203–15. 12960804 10.1097/00000478-200309000-00003

[39] Lin Y Yang H Cai Q. Characteristics and prognostic analysis of 69 patients with pulmonary sarcomatoid carcinoma. Am J Clin Oncol. 2016;39:215–22. 25068469 10.1097/COC.0000000000000101

[40] Hou J Xing L Yuan Y. A clinical analysis of 114 cases of sarcomatoid carcinoma of the lung. Clin Exp Med. 2018;18:555–62. 29987681 10.1007/s10238-018-0517-2

[41] Li X Wu D Liu H Chen J. Pulmonary sarcomatoid carcinoma: progress, treatment and expectations. Ther Adv Med Oncol. 2020;12:1758835920950207. 32922522 10.1177/1758835920950207 PMC7450456

[42] Sim JK Chung SM Choi JH. Clinical and molecular characteristics of pulmonary sarcomatoid carcinoma. Korean J Intern Med. 2018;33:737–44. 29458244 10.3904/kjim.2017.245 PMC6030417

[43] Lococo F Torricelli F Rossi G. Inter-relationship between PD-L1 expression and clinic-pathological features and driver gene mutations in pulmonary sarcomatoid carcinomas. Lung Cancer. 2017;113:93–101. 29110857 10.1016/j.lungcan.2017.09.009

[44] Koss MN Hochholzer L O’Leary T. Pulmonary blastomas. Cancer. 1991;67:2368–81. 1849449 10.1002/1097-0142(19910501)67:9<2368::aid-cncr2820670926>3.0.co;2-g

[45] Yousem SA Wick MR Randhawa P Manivel JC. Pulmonary blastoma. An immunohistochemical analysis with comparison with fetal lung in its pseudoglandular stage. Am J Clin Pathol. 1990;93:167–75. 2301281 10.1093/ajcp/93.2.167

[46] Fishback NF Travis WD Moran CA Guinee DG McCarthy WF Koss MN. Pleomorphic (spindle/giant cell) carcinoma of the lung. A clinicopathologic correlation of 78 cases. Cancer. 1994;73:2936–45. 8199991 10.1002/1097-0142(19940615)73:12<2936::aid-cncr2820731210>3.0.co;2-u

[47] Koss MN Hochholzer L Frommelt RA. Carcinosarcomas of the lung: a clinicopathologic study of 66 patients. Am J Surg Pathol. 1999;23:1514–26. 10584705 10.1097/00000478-199912000-00009

[48] Weissferdt A. Pulmonary sarcomatoid carcinomas: a review. Adv Anat Pathol. 2018;25:304–13. 29912718 10.1097/PAP.0000000000000202

[49] Humphrey PA Scroggs MW Roggli VL Shelburne JD. Pulmonary carcinomas with a sarcomatoid element: an immunocytochemical and ultrastructural analysis. Hum Pathol. 1988;19:155–65. 2449386 10.1016/s0046-8177(88)80343-5

[50] Nappi O Glasner SD Swanson PE Wick MR. Biphasic and monophasic sarcomatoid carcinomas of the lung. A reappraisal of “carcinosarcomas” and “spindle-cell carcinomas”. Am J Clin Pathol. 1994;102:331–40. 8085557 10.1093/ajcp/102.3.331

[51] Attanoos RL Papagiannis A Suttinont P Goddard H Papotti M Gibbs AR. Pulmonary giant cell carcinoma: pathological entity or morphological phenotype? Histopathology. 1998;32:225–31. 9568507 10.1046/j.1365-2559.1998.00378.x

[52] Inomata M Tsuda T Ichikawa T. Efficacy of immune checkpoint inhibitor therapy in patients with pulmonary sarcomatoid carcinoma in clinical practice. Thorac Cancer. 2023;14:1618–23. 37101081 10.1111/1759-7714.14907 PMC10260484

[53] Sun J Jiang Z Shan T. Characteristics and prognostic analysis of 55 patients with pulmonary sarcomatoid carcinoma. Front Oncol. 2022;12:833486. 35592676 10.3389/fonc.2022.833486 PMC9113756

[54] Knuuttila A Jee KJ Taskinen E Wolff H Knuutila S Anttila S. Spindle cell tumours of the pleura: a clinical, histological and comparative genomic hybridization analysis of 14 cases. Virchows Arch. 2006;448:135–41. 16170537 10.1007/s00428-005-0059-3

[55] El-Abassi R Soliman MY Williams F England JD. Whipple’s disease. J Neurol Sci. 2017;377:197–206. 28477696 10.1016/j.jns.2017.01.048

[56] Dolmans RAV Boel CHE Lacle MM Kusters JG. Clinical manifestations, treatment, and diagnosis of tropheryma whipplei infections. Clin Microbiol Rev. 2017;30:529–55. 28298472 10.1128/CMR.00033-16 PMC5355640

[57] Durand DV Lecomte C Cathébras P Rousset H Godeau P. Whipple disease. Clinical review of 52 cases. The SNFMI Research Group on Whipple Disease. Société Nationale Française de Médecine Interne. Medicine (Baltimore). 1997;76:170–84. 9193452 10.1097/00005792-199705000-00003

[58] Schneider T Moos V Loddenkemper C Marth T Fenollar F Raoult D. Whipple’s disease: new aspects of pathogenesis and treatment. Lancet Infect Dis. 2008;8:179–90. 18291339 10.1016/S1473-3099(08)70042-2

[59] Ito M Minamiya Y Kawai H. Intraoperative detection of lymph node micrometastasis with flow cytometry in non-small cell lung cancer. J Thorac Cardiovasc Surg. 2005;130:753–8. 16153924 10.1016/j.jtcvs.2005.05.012

[60] Irish JM Doxie DB. High-dimensional single-cell cancer biology. In: Fienberg HG Nolan GP, Hrsg. High-Dimensional Single Cell Analysis. Springer Berlin Heidelberg; 2014:1–21. 10.1007/82_2014_367 PMC4216808 24671264

[61] Rice TW Bauer TW Gephardt GN Medendorp SV McLain DA Kirby TJ. Prognostic significance of flow cytometry in non-small-cell lung cancer. J Thorac Cardiovasc Surg. 1993;106:210–7. 8393505

[62] Chaft JE Sima CS Ginsberg MS. Clinical outcomes with perioperative chemotherapy in sarcomatoid carcinomas of the lung. J Thorac Oncol. 2012;7:1400–5. 22895138 10.1097/JTO.0b013e3182614856 PMC3632635

[63] Bae H-M Min HS Lee S-H. Palliative chemotherapy for pulmonary pleomorphic carcinoma. Lung Cancer. 2007;58:112–5. 17574296 10.1016/j.lungcan.2007.05.006

[64] Karim NA Schuster J Eldessouki I. Pulmonary sarcomatoid carcinoma: University of Cincinnati experience. Oncotarget. 2018;9:4102–8. 29423107 10.18632/oncotarget.23468 PMC5790524

[65] Reck M von Pawel J Zatloukal P.; BO17704 Study Group. Overall survival with cisplatin-gemcitabine and bevacizumab or placebo as first-line therapy for nonsquamous non-small-cell lung cancer: results from a randomised phase III trial (AVAiL). Ann Oncol. 2010;21:1804–9. 20150572 10.1093/annonc/mdq020 PMC2924992

[66] Blum RH Edmonson J Ryan L Pelletier L. Efficacy of ifosfamide in combination with doxorubicin for the treatment of metastatic soft-tissue sarcoma. The Eastern Cooperative Oncology Group. Cancer Chemother Pharmacol. 1993;31(Suppl 2):S238–240. 8453706

[67] Tomasini P Barlesi F Mascaux C Greillier L. Pemetrexed for advanced stage nonsquamous non-small cell lung cancer: latest evidence about its extended use and outcomes. Ther Adv Med Oncol. 2016;8:198–208. 27239238 10.1177/1758834016644155 PMC4872256

[68] Paredes Mogica JA Reyes Sanchez E Zaragoza Morales DA Pierre-Louis Guillen N Magallanes Maciel ME. Rapidly progressive lung sarcomatoid carcinoma managed with doxorubicin plus ifosfamide and pemetrexed. Case Rep Oncol. 2021;14:1677–81. 35082625 10.1159/000520190 PMC8740224

[69] Hong JY Choi MK Uhm JE. The role of palliative chemotherapy for advanced pulmonary pleomorphic carcinoma. Med Oncol. 2009;26:287–91. 18989796 10.1007/s12032-008-9117-4

[70] Grünert S Jechlinger M Beug H. Diverse cellular and molecular mechanisms contribute to epithelial plasticity and metastasis. Nat Rev Mol Cell Biol. 2003;4:657–65. 12923528 10.1038/nrm1175

[71] Thiery JP. Epithelial-mesenchymal transitions in tumour progression. Nat Rev Cancer. 2002;2:442–54. 12189386 10.1038/nrc822

[72] Bhangu A Wood G Brown G Darzi A Tekkis P Goldin R. The role of epithelial mesenchymal transition and resistance to neoadjuvant therapy in locally advanced rectal cancer. Colorectal Dis. 2014;16:O133–143. 24617665 10.1111/codi.12482

[73] Kim NH Kim HS Li X-Y. A p53/miRNA-34 axis regulates Snail1-dependent cancer cell epithelial-mesenchymal transition. J Cell Biol. 2011;195:417–33. 22024162 10.1083/jcb.201103097 PMC3206336

[74] Chang Y-L Wu C-T Shih J-Y Lee Y-C. EGFR and p53 status of pulmonary pleomorphic carcinoma: implications for EGFR tyrosine kinase inhibitors therapy of an aggressive lung malignancy. Ann Surg Oncol. 2011;18:2952–60. 21409490 10.1245/s10434-011-1621-7

[75] Kaira K Horie Y Ayabe E. Pulmonary pleomorphic carcinoma: a clinicopathological study including EGFR mutation analysis. J Thorac Oncol. 2010;5:460–5. 20107421 10.1097/JTO.0b013e3181ce3e3c

[76] Ushiki A Koizumi T Kobayashi N. Genetic heterogeneity of EGFR mutation in pleomorphic carcinoma of the lung: response to gefitinib and clinical outcome. Jpn J Clin Oncol. 2009;39:267–70. 19155283 10.1093/jjco/hyn155

[77] Fallet V Saffroy R Girard N. High-throughput somatic mutation profiling in pulmonary sarcomatoid carcinomas using the LungCarta^TM^ Panel: exploring therapeutic targets. Ann Oncol Off J Eur Soc Med Oncol. 2015;26:1748–53. 10.1093/annonc/mdv232 25969368

[78] Schrock AB Li SD Frampton GM. Pulmonary sarcomatoid carcinomas commonly harbor either potentially targetable genomic alterations or high tumor mutational burden as observed by comprehensive genomic profiling. J Thorac Oncol. 2017;12:932–42. 28315738 10.1016/j.jtho.2017.03.005

[79] Liu X Jia Y Stoopler MB. Next-generation sequencing of pulmonary sarcomatoid carcinoma reveals high frequency of actionable MET gene mutations. J Clin Oncol. 2016;34:794–802. 26215952 10.1200/JCO.2015.62.0674

[80] Li X Wang D Zhao Q. Clinical significance and next-generation sequencing of Chinese pulmonary sarcomatoid carcinoma. Sci Rep. 2017;7:3947. 28638113 10.1038/s41598-017-04296-2 PMC5479802

[81] Reck M Rodríguez-Abreu D Robinson AG.; KEYNOTE-024 Investigators. Pembrolizumab versus chemotherapy for PD-L1-positive non-small-cell lung cancer. N Engl J Med. 2016;375:1823–33. 27718847 10.1056/NEJMoa1606774

[82] Borghaei H Paz-Ares L Horn L. Nivolumab versus docetaxel in advanced nonsquamous non-small-cell lung cancer. N Engl J Med. 2015;373:1627–39. 26412456 10.1056/NEJMoa1507643 PMC5705936

[83] Topalian SL Taube JM Anders RA Pardoll DM. Mechanism-driven biomarkers to guide immune checkpoint blockade in cancer therapy. Nat Rev Cancer. 2016;16:275–87. 27079802 10.1038/nrc.2016.36 PMC5381938

[84] Postow MA Callahan MK Wolchok JD. Immune checkpoint blockade in cancer therapy. J Clin Oncol. 2015;33:1974–82. 25605845 10.1200/JCO.2014.59.4358 PMC4980573

[85] Kim S Kim M-Y Koh J. Programmed death-1 ligand 1 and 2 are highly expressed in pleomorphic carcinomas of the lung: comparison of sarcomatous and carcinomatous areas. Eur J Cancer. 2015;51:2698–707. 26329973 10.1016/j.ejca.2015.08.013

[86] Vieira T Antoine M Hamard C. Sarcomatoid lung carcinomas show high levels of programmed death ligand-1 (PD-L1) and strong immune-cell infiltration by TCD3 cells and macrophages. Lung Cancer. 2016;98:51–8. 27393506 10.1016/j.lungcan.2016.05.013

[87] Schenk E Boland J Mansfield A Aubry MC Dietz A. Local and systemic immunity predict survival in patients with pulmonary sarcomatoid carcinoma. Med Oncol. 2017;34:140. 28711968 10.1007/s12032-017-1000-8

[88] Pécuchet N Vieira T Rabbe N. Molecular classification of pulmonary sarcomatoid carcinomas suggests new therapeutic opportunities. Ann Oncol. 2017;28:1597–604. 28419182 10.1093/annonc/mdx162

[89] Cimpeanu E Ahmed J Zafar W. Pembrolizumab – emerging treatment of pulmonary sarcomatoid carcinoma: a case report. World J Clin Cases. 2020;8:97–102. 31970174 10.12998/wjcc.v8.i1.97 PMC6962068

[90] Kotlowska MP Rueda AG Olmedo ME. Efficacy of immunotherapy in sarcomatoid lung cancer, a case report and literature review. Respir Med Case Rep. 2019;26:310–4. 30931249 10.1016/j.rmcr.2019.02.017 PMC6409391

[91] Shimoji M Shimizu S Sato K. Clinical and pathologic features of lung cancer expressing programmed cell death ligand 1 (PD-L1). Lung Cancer. 2016;98:69–75. 27393509 10.1016/j.lungcan.2016.04.021

